# Predictive value of 10-year atherosclerotic cardiovascular disease risk equations from the China-PAR for new-onset lower extremity peripheral artery disease

**DOI:** 10.3389/fcvm.2022.933054

**Published:** 2022-10-04

**Authors:** Pengkang He, Fangfang Fan, Chuyun Chen, Bo Liu, Jia Jia, Pengfei Sun, Jianping Li, Jing Zhou, Yan Zhang

**Affiliations:** Department of Cardiology, Peking University First Hospital, Institute of Cardiovascular Disease, Peking University First Hospital, Beijing, China

**Keywords:** lower extremity peripheral artery disease, China-PAR risk equation, risk prediction, Chinese population, ASCVD

## Abstract

Lower extremity peripheral artery disease (LEPAD) is a common and serious health-threatening disease. The aim of this study was to evaluate the predictive value of 10-year atherosclerotic cardiovascular disease (ASCVD) risk equations from the Prediction for ASCVD Risk in China (China-PAR) project for incident LEPAD after 6.75 ± 0.13 years of follow-up. A total of 3,595 Chinese participants without baseline ASCVD or LEPAD from a community-based cohort were enrolled in our study. The mean (interquartile range) baseline 10-year China-PAR ASCVD risk was 4.35% (2.24–8.44%), and the incidence of new-onset LEPAD during 6.75 ± 0.13 years was 4.23%. In univariable logistic regression analysis, 10-year China-PAR ASCVD risk was significantly associated with LEPAD incidence (odds ratio [OR] for each 1% increase in the risk score = 1.06, 95% confidence interval [CI]: 1.03–1.08, *P* < 0.001). After adjusting confounders, the relationship remained significant (OR: 1.09, 95% CI: 1.05–1.1. *P* < 0.001). Participants with the highest risk (≥10%) had significantly increased risk compared to those with the lowest risk (<5%) (OR = 2.65, 95% CI: 1.15–6.07, *P* = 0.022). Further interaction analyses showed no evidence of heterogeneity according to sex, age, body mass index (BMI), smoking, drinking, hypertension, diabetes mellitus, dyslipidemia, renal function, waist circumference, and family history. In conclusion, 10-year China-PAR ASCVD risk independently predicted the risk of new-onset LEPAD in a Chinese community-based population, indicating the importance of polyvascular diseases (PVDs) and the intrinsic interactions of its components.

## Introduction

Lower extremity peripheral artery disease (LEPAD), as an obstructive atherosclerosis disease, is a global health issue, which has caused a huge medical and economic burden worldwide (1). The estimated prevalence of LEPAD without symptoms in the adult population is between 3 and 10% (2). With shifting risk factors and aging population, the burden will be increasing in the future. Patients with LEPAD have 2–6 times the risk in both cardiovascular and cerebrovascular events compared to normal individuals (3).

The prediction model for LEPAD has been established in American populations (4); however, studies focusing on LEPAD risk prediction in the Chinese population have not been conducted yet. As a common subtype of atherosclerotic cardiovascular disease (ASCVD), LEPAD shared similar risk factors with ischemic cardiovascular disease such as hypertension, diabetes, hypercholesterolemia, and smoking. LEPAD with or without symptoms can significantly increase the risk of cardiovascular death (5, 6). Furthermore, previous studies have revealed that LEPAD and other ASCVDs also had similar pathophysiological mechanisms (7). The severity of vascular atherosclerosis in one arterial bed may predict the severity of vascular lesions in other arterial beds (8). Therefore, we speculated that risk score predicting the new-onset of ischemic stroke and acute myocardial infarction may also predict the risk of new-onset LEPAD.

The 10-year ASCVD risk equation from the Prediction for ASCVD Risk in China (China-PAR) project is an effective tool to predict the risk of ASCVD defined as nonfatal acute myocardial infarction or coronary heart disease (CHD) death or fatal or nonfatal stroke in the Chinese population (9). Furthermore, previous studies have developed prediction models using cohorts from decade ago (10, 11). These prediction equations might not be effective enough due to changes in ASCVD risk factors. China-PAR risk equations generate better outcomes because of recent cohort data and the outcome has been validated by two independent cohorts (9).

In the initial study design, the China-PAR study did not include the part of LEPAD risk prediction. Therefore, we performed this longitudinal cohort study to investigate whether 10-year ASCVD risk according to the China-PAR equation predicts incident LEPAD in a Chinese community-based population without LEPAD at baseline with a more than 6-year follow-up.

## Materials and methods

### Study populations

**FIGURE 1:**
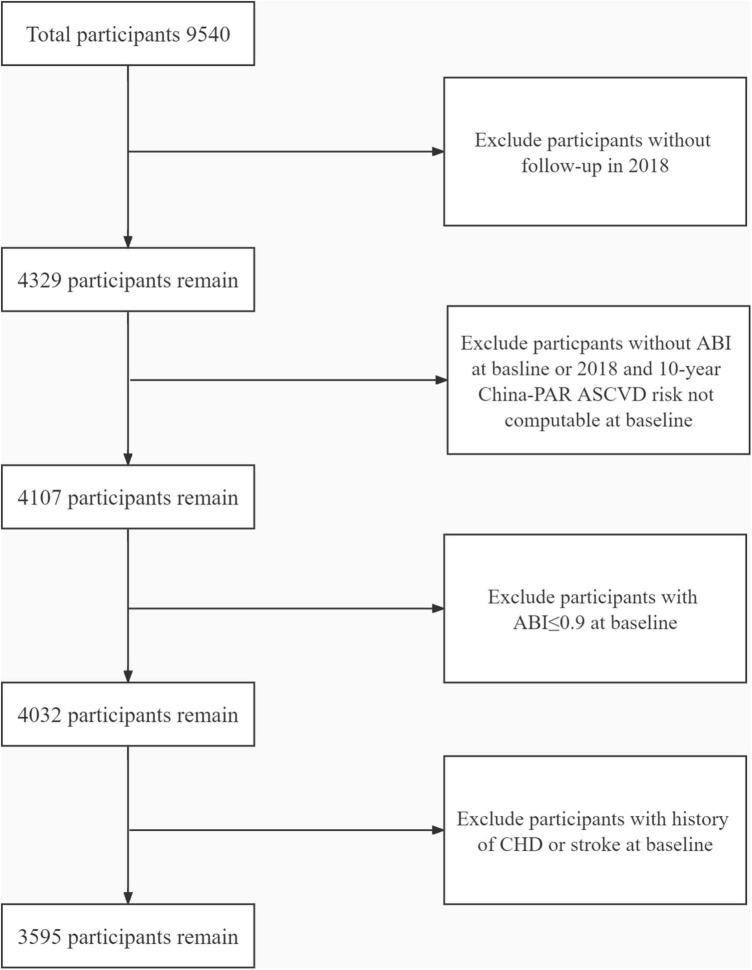
The flowchart of this study. ABI, ankle-brachial index; CHD, coronary heart disease.

The study participants were drawn from an atherosclerosis cohort survey in Gucheng Community and Pingguoyuan Community of Shijingshan District of Beijing, China (12). The baseline survey of this cohort enrolled 9,540 community residents with ≥40 years who had health records in community health centers from December 2011 to April 2012. Of them, 4,329 participants responded to the follow-up study from September to December in 2018. In this study, 4,107 participants were included for having both ankle-brachial index (ABI) results in 2012 and 2018 and computable baseline 10-year China-PAR ASCVD risk. We further excluded participants who have already suffered from stroke and CHD and those with ABI ≤ 0.9 at baseline (Figure 1). Finally, 3,595 participants were enrolled in this analysis. This study was approved by the ethics committee of Peking University First Hospital, and each participant provided written informed consent. We adhered to the principles of the Declaration of Helsinki. The procedures followed were in accordance with institutional guidelines.

### Data collection

The research data were obtained by trained research assistants using standard questionnaires. This basic information questionnaire included social demographic conditions, occupation, education, lifestyle, diet, medical history, medications, and family history. Body mass index (BMI) was calculated by dividing weight (kg) by the square of height (m). Current drinking was defined as drinking once a week or more for more than half a year. Current smoking was defined as smoking one or more cigarettes a day for more than half a year. Waist circumference was defined as the minimal circumference between the inferior margin of the ribcage and the crest of the ileum.

After resting for 5 min in the sitting position, the peripheral (brachial) blood pressure in the sitting position was measured by OMRON (Kyoto, Japan) HEM-7117 electronic sphygmomanometer. A total of three consecutive blood pressure measurements were performed on the participants’ right upper arm, with an interval of at least 1 min. Each participant’s systolic blood pressure and diastolic blood pressure used in the data analysis were the average of the three measurements.

After overnight fasting, a venous blood sample was obtained from each participant’s forearm. Blood samples were used for the measurement of fasting blood glucose, the standard 75 g oral glucose tolerance test, total cholesterol, low-density lipoprotein cholesterol, high-density lipoprotein cholesterol, triglycerides, and creatinine. Roche (Basel, Switzerland) C8000 Automatic Analyzer was applied for laboratory tests at baseline.

The blood pressure (BP) of both upper (brachial artery) and lower (ankles) limbs of participants in supine position over 5 min was collected *via* a BP-203RPE III machine (Omron Healthcare, Japan), and ABI results were automatically calculated by the machine. New-onset LEPAD was defined as at least one ABI value of ≤0.9 at the follow-up survey in 2018.

Hypertension was defined as any self-reported history of hypertension, systolic BP ≥ 140 mm Hg, diastolic BP ≥ 90 mm Hg, or use of any antihypertensive drugs. Diabetes mellitus was defined as any self-reported history of diabetes, or fasting blood glucose greater than 7 mmol/L, or 2-h blood glucose higher than 11.1 mmol/L in oral glucose tolerance test, or any history of hypoglycemic drugs usage. The definition of hyperlipidemia was that patients had a history of using lipid-lowering drugs or met one of the following conditions: triglyceride > 1.70 mmol/L (150 mg/dl), total cholesterol ≥ 5.18 mmol/L (200 mg/dl), low-density lipoprotein cholesterol ≥ 3.37 mmol/L (130 mg/dl), and high-density lipoprotein cholesterol < 1.04 mmol/L (40 mg/dl). Renal function was divided into three groups by estimated glomerular filtration rate (eGFR): group 1: ≥ 90 ml/min/1.73 m^2^, group 2: 60 to <90 ml/min/1.73 m^2^, and group 3: <60 ml/min/1.73 m^2^. eGFR was calculated by the following equation (13):

$$\text{eGFR}=141\times \min\left(\frac{\text{Scr}}{\text{K}},1\right)\,\alpha \times \max\left(\frac{\text{Scr}}{\text{K}},1\right)-1.209\times 0.993\,\text{Age}\times 1.018\,\left(\mathrm{if}\,\mathrm{female}\right)$$

where Scr is serum creatinine (mg/dl); K is 0.7 for women and 0.9 for men; α is -0.329 for women and -0.411 for men; min indicates the minimum of Scr/K or 1; and max indicates the maximum of Scr/κ or 1.

### Statistical analysis

Data were expressed as mean ± standard deviation (SD) for continuous variables and *n* (%) for categorical variables. ANOVA or Kruskal–Wallis rank test was used for the comparison of differences among different groups for continuous data with normal distribution or skewed distribution appropriately. For categorical variables, the chi-square test was adopted.

China-PAR risks were calculated using these parameters including sex, age, systolic blood pressure, total cholesterol, high-density lipoprotein cholesterol, waist circumference, current smoking or not, diabetes or not, anti-hypertensive treatment or not, geographic regions (urban or rural), and family history of ASCVD (5).

Univariable and multivariable logistic regression was used to evaluate the association of China-PAR risk and the odds ratio (OR) of new-onset LEPAD. Covariates included sex, age, BMI, baseline ABI, current smoking and drinking, hypertension, diabetes mellitus, dyslipidemia, and eGFR groups. Interactive analyses were further performed to test possible heterogeneities in different subgroups according to the covariates.

To compare the ability of China-PAR to predict new-onset LEPAD with traditional risk factors, we established two models. We further compared the area under the curve (AUC) of model 1 and model 2 to determine which model has better predictive power.

All analyses were performed using Empower^®^ (X&Y Solutions, Boston, MA, USA)^1^ and R.^2^ A *P*-value of <0.05 (two-sided) was considered statistically significant for all tests.

## Results

### Participants’ characteristics

The demographic characteristics of all participants at baseline are shown in Table 1. Participants were 55.05 ± 7.60 years old, 33.32% of them were men, and the mean (SD) ABI was 1.11 ± 0.08. The median 10-year China-PAR ASCVD risk was 4.35% (2.24–8.44%). Of all participants, 40.81% had hypertension, 18.86% had diabetes, and 68.90% was combined with dyslipidemia. According to China-PAR risks of all participants, 55.16% (1,983 of 3,595) had the lowest 10-year ASCVD risk (<5%), and the proportion of participants with middle (5 to <10%) and highest risks (≥10%) was 25.79% (927 of 3,595) and 19.05% (685 of 3,595), respectively. Three groups showed distinguished differences in all variables except for total cholesterol. Higher risk group patients were older (57.20 ± 6.46 in the middle-risk group and 62.22 ± 8.18 in the highest risk group) and more males (50.05% in the middle-risk group and 77.81% in the highest risk group, while 10.14% in the lowest risk group) and showed higher percentage in ASCVD risk factors like current smoking and drinking and combined diseases. Current smoking in the highest risk group, the middle-risk group, and the lowest risk group was 36.20, 28.80, and 5.80%, respectively. Proportions of hypertension in the three groups were 81.46% in the highest risk group, 50.81% in the middle-risk group, and 22.09% in the lowest risk group. Proportions of diabetes in the three groups were 46.13% in the highest risk group, 26.21% in the middle-risk group, and 6.00% in the lowest risk group. They had poorer control of blood pressure and poorer renal function than low-risk patients. eGFR in three groups was 88.90 ± 12.81 ml/min/1.73 m^2^, 94.19 ± 11.47 ml/min/1.73 m^2^, and 100.20 ± 9.74 ml/min/1.73 m^2^ (highest group, middle-risk group, and lowest group, respectively). Meanwhile, higher proportions of medication usage were also found in higher risk groups. Proportions of antihypertensive drugs in three groups were 49.34, 30.79, and 12.39% (highest group, middle-risk group, and lowest group, respectively). Proportions of hypoglycemic drugs were 15.96, 9.64, and 1.87%, respectively.

**TABLE 1:** Baseline characteristics of all eligible participants.

Variables	Total	10-year ASCVD risk (%)		
		<5	5–<10	≥10
*N*	3,595	1,983	927	685
Males, *n* (%)	1,198 (33.32%)	201 (10.14%)	464 (50.05%)	533 (77.81%)
Age (years)	55.05 ± 7.60	51.56 ± 5.49	57.20 ± 6.46	62.22 ± 8.18
Waist (cm)	81.95 ± 8.40	78.77 ± 7.58	85.11 ± 7.93	86.91 ± 7.15
Body mass index (kg/m^2^)	25.85 ± 3.27	25.19 ± 3.14	26.64 ± 3.41	26.67 ± 3.04
Current smoking, *n* (%)	630 (17.52%)	115 (5.80%)	267 (28.80%)	248 (36.20%)
Current drinking, *n* (%)	837 (23.29%)	257 (12.97%)	311 (33.55%)	269 (39.27%)
Systolic blood pressure (mmHg)	131.35 ± 15.68	124.60 ± 12.71	135.43 ± 13.46	145.37 ± 15.03
Diastolic blood pressure (mmHg)	75.00 ± 9.55	72.86 ± 8.49	76.62 ± 9.22	78.98 ± 11.06
Total cholesterol (mmol/L)	5.34 ± 0.99	5.36 ± 0.96	5.37 ± 1.04	5.27 ± 0.99
High-density lipoprotein (mmol/L)	1.46 ± 0.38	1.56 ± 0.38	1.37 ± 0.34	1.28 ± 0.32
eGFR (ml/min/1.73 m^2^)	96.50 ± 11.72	100.20 ± 9.74	94.19 ± 11.47	88.90 ± 12.81
Combined diseases, *n* (%)				
Hypertension	1,467 (40.81%)	438 (22.09%)	471 (50.81%)	558 (81.46%)
Diabetes	678 (18.86%)	119 (6.00%)	243 (26.21%)	316 (46.13%)
Dyslipidemia	2,477 (68.90%)	1,292 (65.15%)	686 (74.00%)	499 (72.85%)
Medications, *n* (%)				
Antihypertensive	860 (24.23%)	242 (12.39%)	282 (30.79%)	336 (49.34%)
Hypoglycemic	235 (6.55%)	37 (1.87%)	89 (9.64%)	109 (15.96%)
Lipid-lowering	242 (6.86%)	96 (4.93%)	85 (9.40%)	61 (9.01%)
10-year ASCVD risk, *n* (%)	4.35 (2.24–8.44)	2.43 (1.50–3.49)	6.98 (5.95–8.27)	14.19 (11.81–18.50)

Data are expressed as mean ± standard deviation or median (interquartile range) as appropriate for continuous variables and *n* (%) for categorical variables.

ASCVD, atherosclerotic cardiovascular disease; ABI, ankle–brachial index; eGFR, estimated glomerular filtration rate.

### Associations of 10-year China-PAR atherosclerotic cardiovascular disease risk and new-onset lower extremity peripheral artery disease

After 6.75 ± 0.13 years of follow-up, 152 (4.23%) participants developed new-onset LEPAD, and those with 10-year China-PAR ASCVD risk of 10% had the highest risk (7.88%) of new-onset LEPAD (Table 2).

**TABLE 2:** The relationships between China-PAR risk score and new-onset LEPAD.

Variables	Total	10-year ASCVD risk (%)			*P*-value
		<5%	5–<10%	≥10%	
*N*	3,595	1983	927	685	
ABI in 2012	1.11 ± 0.08	1.09 ± 0.08	1.12 ± 0.08	1.13 ± 0.08	< 0.001
ABI in 2018	1.08 ± 0.10	1.08 ± 0.09	1.09 ± 0.09	1.09 ± 0.12	0.023
New-onset LEPAD, %	152 (4.23%)	72 (3.63%)	26 (2.80%)	54 (7.88%)	< 0.001

ASCVD, atherosclerotic cardiovascular disease; ABI, ankle–brachial index; LEPAD, lower extremity peripheral artery disease.

**FIGURE 2:**
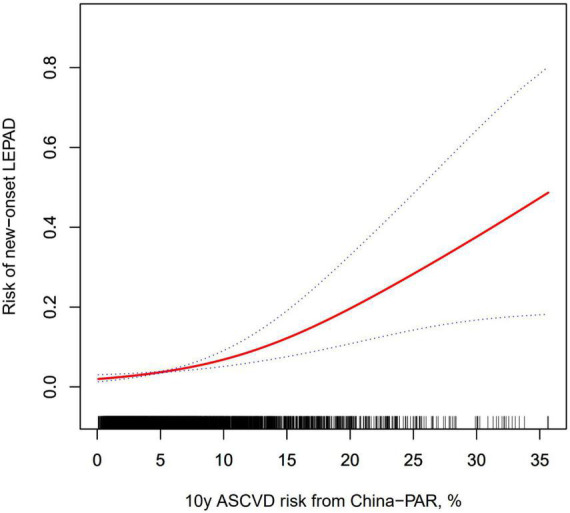
Penalized thin plate regression spline for the association of new-onset LEPAD and 10-year China-PAR ASCVD risk. The solid line represents the smoothing curve; the dashed lines represent the 95% confidence interval. The smoothing curve was adjusted for sex, BMI, age, baseline ABI, current smoking and drinking status, diabetes mellitus, hypertension, and dyslipidemia. ASCVD, atherosclerotic cardiovascular disease; BMI, body mass index; LEPAD, lower extremity peripheral artery disease.

Figure 2 shows the penalized thin plate regression spline of 10-year China-PAR ASCVD risk and new-onset LEPAD adjusted for sex, BMI, age, baseline ABI, current smoking and drinking status, diabetes mellitus, hypertension, and dyslipidemia, which showed that the risk of LEPAD increased with the elevation of 10-year China-PAR ASCVD risk with no inflection point.

Table 3 displays the results of logistic regressions for the association of 10-year China-PAR ASCVD risk with new-onset LEPAD. China-PAR risk was significantly associated with the risk of new-onset LEPAD (for each 1% increase in China-PAR risk: OR = 1.06, 95% CI: 1.03–1.08, *P* < 0.001). After adjusting various confounders, the association still remained significant (for each 1% increase in China-PAR risk: OR = 1.10, 95% CI: 1.04–1.15, *P* < 0.001). Similarly, the OR of new-onset LEPAD was highest in participants with China-PAR risk of ≥10% compared to those with China-PAR risks of <10% (OR = 3.29, 95% CI: 1.82–5.92, *P* < 0.001) and <5% (OR = 2.65, 95% CI: 1.15–6.07, *P* = 0.022). We also performed regression analyses adjusting variables not used to calculate the China-PAR risk in Model III, which reached similar results.

**TABLE 3:** Odds ratios of new-onset LEPAD by 10-year China-PAR ASCVD risk.

Variables	Crude model		Adjusted model I		Adjusted model II	
	OR (95% CI)	*P*-value	OR (95% CI)	*P*-value	OR (95% CI)	*P*-value
**10-year ASCVD risk, per 1%**						
	1.06 (1.03, 1.08)	<0.001	1.11 (1.07, 1.15)	<0.001	1.09 (1.05, 1.12)	<0.001
**10-year ASCVD risk groups, %**						
<5	1.00		1.00		1.00	
5–<10	0.77 (0.49, 1.21)	0.251	1.05 (0.62, 1.76)	0.868	0.93 (0.56, 1.54)	0.783
≥10	2.27 (1.58, 3.27)	<0.001	4.15 (2.27, 7.59)	<0.001	3.36 (2.02, 5.58)	<0.001
<10	1.00		1.00		1.00	
≥10	2.46 (1.74, 3.46)	<0.001	4.03 (2.43, 6.69)	<0.001	3.48 (2.23, 5.42)	<0.001

CI, confidence interval; OR, odds ratio; LEPAD, lower extremity peripheral artery disease; ASCVD, atherosclerotic cardiovascular disease; eGFR, estimated glomerular filtration rate.

Model I: adjusted for baseline ABI, sex, and age.

Model II: adjusted for variables not used to calculating the China-PAR risk including baseline ABI, BMI, current drinking, hypertension, dyslipidemia, eGFR groups, antihypertensive agents, lipid-lowering agents, and hypoglycemic agents.

### Subgroup analyses

**FIGURE 3:**
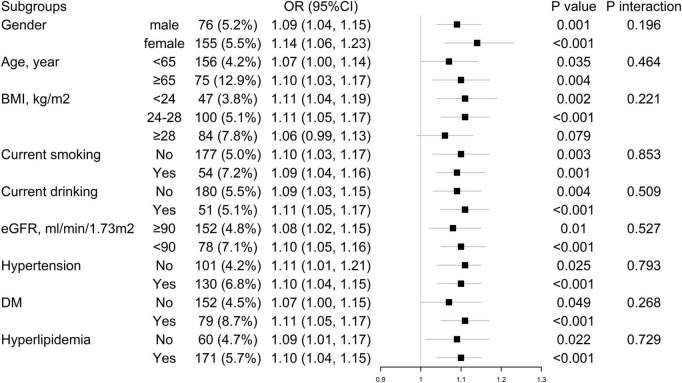
Subgroup and interaction analyses for the association of new-onset LEPAD and 10-year China-PAR ASCVD risk. ASCVD, atherosclerotic cardiovascular disease; BMI, body mass index; CI, confidence interval; DM, diabetes mellitus; OR, odds ratio; eGFR, estimated glomerular filtration rate; LEPAD, lower extremity peripheral artery disease.

Stratification and interaction analyses are shown in Figure 3. Subgroup analysis failed to show significant heterogeneity according to sex, age (<65 or ≥65 years old), BMI (<24, 24–28, or ≥28 kg/m^2^), the status of smoking and drinking, hypertension, diabetes mellitus, dyslipidemia, and renal function (eGFR ≥ 90 ml/min/1.73 m^2^ or < 90 ml/min/1.73 m^2^).

## Discussion

The main finding of our study is that 10-year China-PAR ASCVD risk was associated with the OR of new-onset LEPAD in this Chinese community-based population. Individuals with higher 10-year China-PAR ASCVD risks are more prone to suffer from LEPAD in the future. The ORs of new-onset LEPAD were as high as 3.29 times in participants with China-PAR risk of ≥10% compared to those with China-PAR risk of <10%. A 10-year ASCVD risk calculated by the China-PAR equation can also be taken as a useful tool in the prediction of new-onset LEPAD.

In recent years, much attention was paid to polyvascular disease (PVD), which is a coexisting status of multiple vascular comorbidities including cerebrovascular disease, coronary artery disease, and LEPAD, and is defined as “clinically significant” atherosclerotic disease in at least two major arterial beds in the 2017 European Society of Cardiology guidelines (14). PVD affects about 20% of the population with atherosclerotic disease, and all the constituents of it share similar risk factors and pathological mechanism with each other. Previous studies have proven that PVD would gravely worsen a patient’s clinical outcomes by increasing the possibility of future cardiovascular events and the need of intensive interventions (15, 16). However, LEPAD, especially asymptomatic type, did not draw enough attention of clinicians (17), and few studies evaluated the relationship between the risk of LEPAD and other types of PVD in a longitudinal design. As far as we know, this study is the first to explore the predicting value of 10-year ASCVD (e.g., stroke and myocardial infarction) risk for new-onset LEPAD in the Chinese population, and proved that participants with high risks of ASCVD were also vulnerable to LEPAD.

The China-PAR equation, used in this study to calculate the 10-year risk of ASCVD, was constructed and validated based on data of several large cohorts with as long as 12 years of follow-up in China. The 10-year ASCVD risk predicted by the China-PAR equation was confirmed to be close to the actual incidence of ASCVD, which means a high level of calibration, and this model has also a good performance of discrimination (5). In subsequent studies, China-PAR was also applied to predict the severity of coronary artery disease and the risk of cerebrovascular events in different populations, all of which reflected good performance (18–20). In addition, this study demonstrated that the China-PAR risk equation has a good value in predicting new-onset LEPAD as well as in ASCVD and may provide a support for clinical decision-making.

Regarding the mechanism underlying this result, the component of PVD had a similar pathophysiological basis, and LEPAD was found to be highly associated with traditional CVD risk factors in previous studies including current smoking, diabetes, hypertension, and hypercholesterolemia (21, 22). However, differences still existed. The impact of smoking on LEPAD is significantly stronger than that on stroke and/or myocardial infarction (2, 23, 24). Previous studies have also tried to establish prediction models for LEPAD, whereas most of these models were based on a small number of risk factors including age, smoking, race, sex, pulse pressure, and total cholesterol (25, 26), and none of such studies is carried out in China till present (27). Different from the models in previous studies, the 10-year China-PAR ASCVD risk prediction model was constituted by more comprehensive parameters such as region and waist circumference, which was not included in other ones. Moreover, we found no heterogeneity regarding the predictive value of 10-year China-PAR ASCVD risk in different subgroup populations, which indicates the China-PAR risk equation was robust and had an independent prediction value for LEPAD.

Yet, this study still has several limitations. First, the conclusions of our study are based on a community-based population in Beijing with normal ABI at baseline. Whether this conclusion can be extended to other populations remains to be verified. Second, the definition of LEPAD was obtained through ABI at two time points without a clinical diagnosis. The accuracy of LEPAD prediction might be enhanced by multiple ABI results through the follow-up. However, previous studies have also adopted this definition using ABI (28). Third, whether a 10-year China-PAR ASCVD risk could be implemented in the prediction of atherosclerosis disease in other peripheral artery beds (e.g., renal artery) still remains unclear.

In conclusion, 10-year ASCVD risk calculated by the China-PAR equation was independently associated with the risk of new-onset LEPAD in the Chinese community-based population, providing a simple and effective tool to predict the future risk of LEPAD. This study also indicates the importance of the concept of PVD and the intrinsic interactions of its components.

## Data availability statement

The original contributions presented in this study are included in the article/supplementary material, further inquiries can be directed to the corresponding authors.

## Ethics statement

The studies involving human participants were reviewed and approved by Peking University First Hospital Ethics Committee. The patients/participants provided their written informed consent to participate in this study.

## Author contributions

All authors listed have made a substantial, direct, and intellectual contribution to the work, and approved it for publication.

## Footnotes

1
www.empowerstats.com

2
http://www.R-project.org
